# Three-step optimization based on a multi-model of rural tourism sites

**DOI:** 10.1371/journal.pone.0332878

**Published:** 2025-09-24

**Authors:** Qi Li, Mengting Ai, Jing Luo, Yaru Sun, Lingling Tian

**Affiliations:** 1 College of Urban and Environmental Sciences, Central China Normal University, Wuhan, Hubei, China; 2 Hubei Institute of Economic and Social Development, Central China Normal University, Wuhan, Hubei, China; Linyi University, CHINA

## Abstract

Rural tourism is a vital facet of rural revitalization and balanced urban-rural development efforts. As socio-economic development and policy systems change, the tourism demand market progressively transitions from urban to rural areas. This study, based on ‘quantity-location-capacity’ principles, integrates actual environmental conditions and employs location-allocation and algorithmic models to enhance the accessibility of rural tourism sites in the metropolitan fringe area of Wuhan. The results show: ① Wuhan’s distribution of rural tourism destinations is uneven, with the suburbs demonstrating significantly higher composite accessibility compared to the central urban area; ② Based on this experiment’s results and the costs of optimization, there are eight new potential tourist locations; ③ The disparity in accessibility to Wuhan’s tourist destinations has been reduced, resulting in enhanced tourism efficiency and equity. This study can offer recommendations for rural tourism planning and development strategies and provide references for academic research methods.

## Introduction

In October 2022, the 20^th^ National Congress of the Communist Party of China proposed to actively focus on agricultural production, promote a bountiful harvest and income increase for farmers, and aim to achieve comprehensive rural revitalization [1]. Rural tourism, one of many ways to achieve rural revitalization, aids in stimulating the rural economy, promotes the modernization of agriculture, and improves the production and living environment of rural areas [2–4]. As concepts like leisure and ecology gain popularity, urban residents are increasingly inclined to choose green leisure rural tourism. In the development of rural tourism across various regions, areas surrounding cities are primary locations for rural tourism destinations. As the tourism industry develops and consumerism rises, the demand for rural ecological leisure tourism around cities increases, particularly in the peripheral areas of large cities [5]. These areas benefit from a large market of potential tourists, accessible transportation links between urban and rural areas, and striking landscape contrasts [6].

As rural tourism has developed, research in this area has progressively intensified. Current research on rural tourism is abundant in China, mainly focusing on the sustainable development and social impact of rural tourism destinations [7], spatial layout and its influencing factors [8], spatial evolution characteristics [9], driving mechanisms [10], and the public’s perception and satisfaction with rural tourism destinations [11]. Internationally, research focuses on the elements of rural cultural tourism, community participation, and the impact of policy and culture on the sustainable development of rural tourism [12–14]. However, despite existing research’s progress in qualifying and quantifying aspects of rural tourism destinations, there is a lack of practical research on the optimization of rural tourism destinations. Accessibility is an effective evaluation index for infrastructure development. Analyzing factors like convenience of transportation mobility is important for understanding the development of rural tourism [15,16]. In fact, accessibility is a multidimensional concept that involves many factors, such as road traffic conditions, the number of opportunities for resources, the distribution of social groups, individual supply and demand opportunities, and modes of travel [17–19]. Improving the accessibility of rural tourism and tourist destinations can help promote regional economic development, accelerate rural revitalization, and meet the tourism needs of residents. Therefore, researching rural tourism accessibility contributes to the overall efficiency and equity of tourism.

This study aims to optimize the efficiency and equity of rural tourism accessibility. The objective is to apply site selection optimization theory and methods to rural tourism development, focusing on spatial accessibility and supply-demand balance. This approach aims to enrich and broaden the practical applications of site selection optimization within rural tourism contexts.

## Literature review

### Current status of facility site selection

Facility site selection entails the strategic determination of optimal locations for a set of new facilities within a defined spatial boundary. The purpose is to maximize the spatial distribution of the facilities’ services and societal benefits. Internationally, scholarly research on facility site selection has spanned many years. In 1929, Weber [20] formalized the concept of facility site selection, identifying it as an issue in need of optimization. In studies related to site selection for tourism projects, scholars have established an evaluation index system for tourism destinations, utilizing Geographic Information System (GIS) technology to conduct both single-factor and comprehensive assessments of the evaluation factors. This approach isolates the most suitable areas for development. Fang [21] used elements such as source region location, transportation, temperature, air quality, topography, water systems, and tourist landscapes to evaluate indicators based on the users’ demand preferences. They were able to conduct spatial site selection for villages with distinctive retirement characteristics in the northern Zhejiang province region of China. Wang [22] assessed the suitability of site selection for developing shoreline sports and leisure tourism products on Haitan Island in Fujian Province, focusing on four main factors: geographical climate, environmental constraints, land development and construction conditions, as well as connectivity with cities and scenic areas. Therefore, it is necessary to optimize and select tourism destinations based on both natural and cultural factors. Moreover, as previously mentioned, facility optimization incorporates the surrounding environment, traffic conditions, and population distribution into its calculations.

The idea of accessibility originally stemmed from von Thünen’s agricultural location theory and Weber’s industrial location theory [23] and was used to measure the degree of difficulty with which various nodes interacted and overcame spatial barriers within a regional transportation network. As the application of accessibility and more sophisticated models increases, researchers have developed an innovative accessibility measurement method from the gravity model——the Two-step Floating Catchment Area method (2SFCA) [24]. This method accounts for both the supply and demand capabilities of facilities. It yields more accurate and operable results, and thus has been widely applied and developed. The frequency with which scholars use the 2SFCA and its improved models to measure the accessibility of tourist destinations has gradually increased over time [25,26]. This study is based on the accessibility calculated by the 2SFCA method and will be used to perform subsequent studies.

### Models for optimizing the efficiency and equality of accessibility

“Facility location” refers to the process of determining the best positions for new facilities within a certain spatial scope. This is done to optimize the spatial distribution of facilities, maximizing the comprehensive benefits for both service functionality and societal needs. As location theory has advanced, scholars have developed a series of mathematical models, including the P-median model, the coverage model, the capacity-constrained model, and the minimax mode [27–29]. Additionally, research objectives have shifted from a single focus on efficiency to a balance between equity and efficiency. The available research methods have evolved from basic location-allocation models to improved models with multiple optimization objectives. These models are applied in the research of medical facilities [30], educational facilities [31], elderly care facilities [32], and park landscapes [33]. For example, Tian [34] examined hospital location and capacity optimization through an enhanced two-step method focused on spatial accessibility, aiming to maximize patients’ access to medical services. Cai [35] used the Gauss Two-step Floating Search method to study the accessibility of educational facilities, based on the supply and demand relationship, and adopted an improved P-median model to optimize the spatial layout. Regarding the optimization for facilities, other scholars have also proposed an abundance of models. Weng [36], based on the analysis of landscape pattern and accessibility of three streets in Shenzhen City, used the k-means clustering algorithm and particle swarm optimization algorithm for site selection optimization. Ai [37] used the Particle Swarm Optimization algorithm (PSO) to optimize the location and capacity determination of charging stations. In summary, location-allocation models have been developed to optimize the spatial distribution and capacity of facilities [38–40]. Hence, the research methods for facility optimization have evolved from basic location-allocation models to improved models with multiple optimization objectives.

This paper is dedicated to the spatial reconstruction of rural tourism in rapidly urbanizing areas. Its core value lies in systematically alleviating the supply-demand mismatch caused by urban-rural population flows through a three-stage optimization of “quantity-location-capacity.” Therefore, this paper focuses on rural tourism destinations in Wuhan City, utilizing the P-median model, maximum coverage model, and particle swarm optimization to identify potential site locations while considering environmental contexts. A genetic algorithm is then applied to optimize the capacity of each new site, forming a strategic plan for rural tourism development in Wuhan. This provides a basis for future transportation network expansion and the establishment of additional rural tourism destinations.

## Methodology

### Study area

Wuhan, one of China’s largest cities, is located at 113°41′-115°05′E and 29°58′-31°22′N and spans 8569.15 km². Wuhan consists of seven municipal central districts and six suburban districts. The central and suburban areas account for 11.14% (955.15 km²) and 88.86% (7614.00 km²), respectively. More than half of Wuhan’s population (51.94%, 6.4 million) work and live in the central districts. In terms of tourism resources, suburbs account for a total of 64.91% of the overall 3A and scenic spots in Wuhan, possessing abundant natural and cultural resources. With the rising pressure of modern life and increasing level of consumption, people are changing their means of travel, from long-distance and high-cost outbound travel to short-distance and low-cost travel around the countryside. Therefore, the suburban area is ideal for optimizing rural tourism accessibility from a supply-demand perspective. The methodological design applies to metropolitan fringe areas with similar characteristics of urban-rural population mobility and development patterns.

### Data collection and pretreatment

This study primarily utilizes rural tourism sites data, transportation network data, population data, and land use data. The basic dataset is displayed in Fig 2.

**Fig 1:**
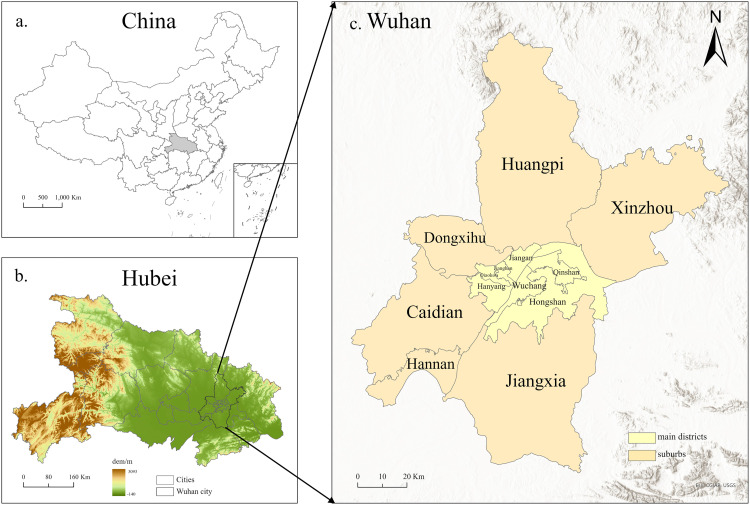
The locations of Wuhan City and Hubei Province in China. (Free vector and raster map data @ naturalearthdata.com.).

Rural tourism sites data was derived from the Point of Interest (POI) data from AMAP and was further supplemented by the Cultural Tourism Bureau and the Agriculture and Rural Affairs Bureau of Wuhan. We scoured a total of 1664 rural tourism sites within six Wuhan suburbs (Dongxihu, Hannan, Caidian, Jiangxia, Huangpi, and Xinzhou). This included ten different venue types (pickling garden, fishing park, vacation village, green park, family farm, scenic area, campsite, Agri turismo, eco-agricultural garden, and memorial hall). After obtaining precise geographic coordinates, the collected data were processed and integrated by removing overlapping attractions, attractions under construction, and closed attractions. Ultimately, a total of 1,046 rural tourism sites were sorted. Transportation network data was sourced from OpenStreetMap and processed in ArcGIS, following the *Technical Standard for Highway Engineering* (JTG B01-2014). Since the current mode of travel for tourists in Wuhan is mainly self-driving and self-service travel, the traffic scenario of this study is set to “driving mode”.

Population data was sourced from the Geographic Census Data of Wuhan, with the community as the basic unit. We obtained community population point data and applied them as representative demand points, using the “Feature to Point” tool in ArcGIS. Land use data was sourced from the SinoLC-1, established by the National Laboratory of Information Engineering of Surveying, Mapping, and Remote Sensing at Wuhan University in 2023, with a resolution of 1 meter.

### Optimization models and methods

This study’s framework, the connotation of its research method, and its related parameter settings are illustrated in Fig 3. The origin of the research method and the formula are shown in Table 1.

**Table 1:** Table of formula, parameter meanings, and the origin of research methods.

Model	Formula		Represent	Origin
2SFCA	$\begin{array}{c} A_{i}^{F}=\sum_{j\in \left\{d_{ij}\leq d_{\text{0}}\right\}}R_{j}*f\left(d_{ij}\right) \end{array}$ $R_{j}=\frac{S_{j}}{\sum_{i\in \left\{d_{ij}\leq d_{\text{0}}\right\}}D_{i}*\;f\left(d_{ij}\right)}$		$A_{i}^{F}$: the accessibility of demand point *i*; $R_{j}$: the supply-demand ratio of supply point *j*; $S_{j}$: the supply scale of supply point *j*; ${𝐃}_{𝐢}$: the demand scale of demand point *i*; $d_{ij}$: the distance between demand point *i* and supply point *j*; $d_{0}$: the search radius.	Radke applied this model to the research on measuring the accessibility of public facilities [24], and it was improved by Luo [46].
K-means	$\begin{array}{c} SSE=\sum_{i}^{n}\sum_{m\;\in C_{i}}{\left(m-M_{i}\right)}^{\text{2}} \end{array}$		**SSE**: the sum of squared errors; ***n***: the number of clusters, ***Ci***: the *i* cluster; ***M***_***i***_: the cluster center of cluster *Ci*. ***(m − Mi)***^***2***^: the Euclidean distance from the *m* point in cluster *Ci* to the cluster center *Mi*.	In 1967, MacQueen first proposed the this model [47].
P-median	$\begin{array}{c} Min\sum_{i}\sum_{j}w_{i}d_{ij}M_{ij} \end{array}$	$M_{ij}\leq N_{j},\forall i,j$ $\sum_{j}M_{ij}=\text{1},\forall i$ $\sum_{j}N_{j}=P,\forall j$ $M_{ij},N_{j}\in \left\{\text{0},\text{1}\right\},\forall ij$	$w_{i}d_{ij}$: the weighted distance between demand point *i* and facility point *j.* When candidate facility point *j* is selected, $N_{j}\;$is assigned a value of 1, otherwise it is assigned a value of 0; when facility point *j* serves demand point *i*, $M_{ij}\;$is assigned a value of 1, otherwise it is 0.	In 1964, Hakimi, based on the study of the optimal location of network centers, proposed this model [35].
MCM	$\begin{array}{c} Max\sum_{i}w_{i}M_{i} \end{array}$	$\sum_{j}N_{j}\leq M_{i},\forall i,j$ $\sum_{j}N_{j}=P,\forall j$ $d_{ij}\leq d_{\text{0}},\forall i,j$ $M_{i},N_{j}\in \left\{\text{0},\text{1}\right\},\forall ij$	$w_{i}\;$**: **the weight of demand point *i*; When demand point *i* is within the service range, *Mi* is assigned a value of 1, otherwise it is 0. When candidate facility point *j* is selected, *Nj* is assigned a value of 1, otherwise it is 0.	This model was proposed by Church in 1974 [43].
PSO	MATLAB toolbox code			In 1995, Kennedy and Eberhart proposed this model [48].
GA	MATLAB toolbox code			In 1975, Professor J. Holland of the United States based this model on natural laws such as biological inheritance, mutation, and natural selection [49].

**Fig 2:**
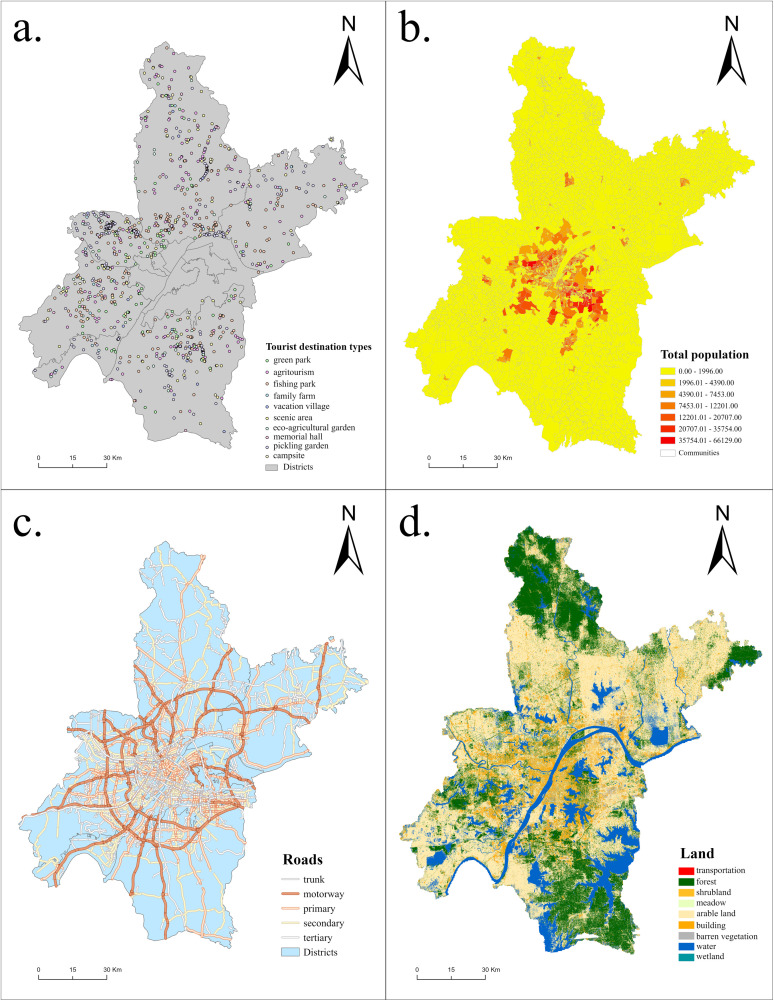
Data map of Wuhan City (a. Tourist destination types, b. Population, c. Roads, and d. Land).

**Fig 3:**
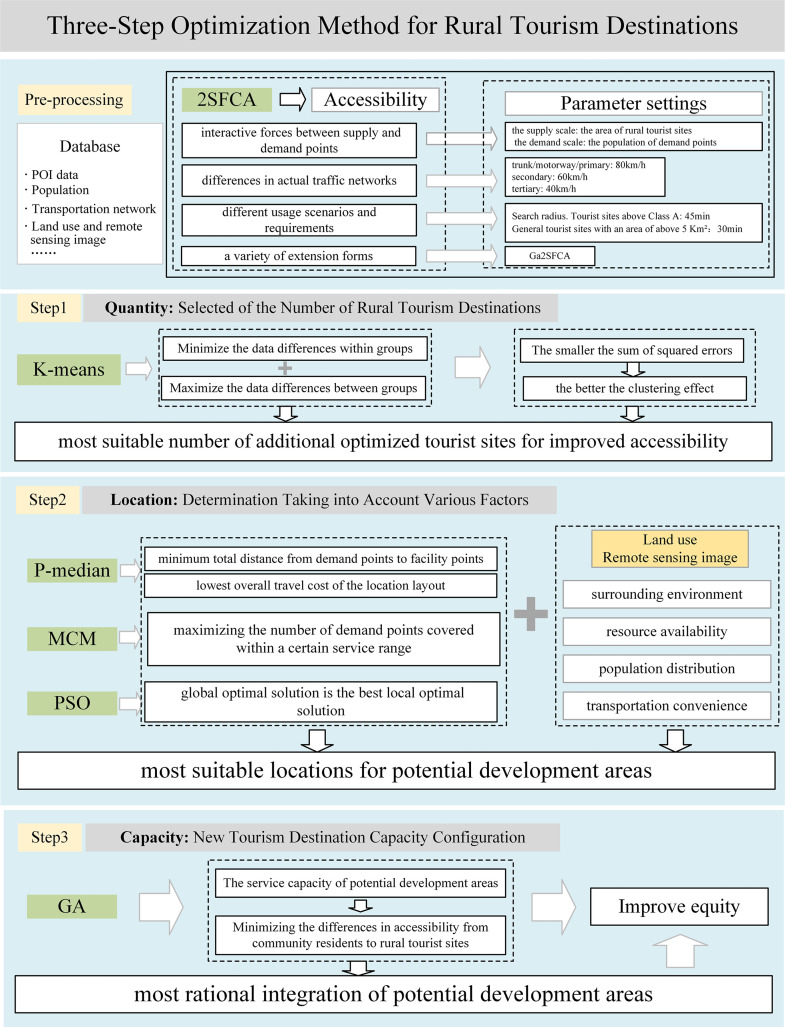
Research framework and parameter settings for the optimization of rural tourism.

#### Accessibility calculation and new land quantity model.

2SFCA accounts for the interactive forces between supply and demand points. Depending on different usage scenarios and requirements, these interactive forces can adopt various forms, such as gravity decay, Gaussian functions, and power functions [41]. Rural tourism demand exhibits a nonlinear decay with increasing distance, and the Gaussian function can more accurately model this decay characteristic: slow decay at short distances, accelerated decay at medium distances, and slowed decay again at long distances. Compared to traditional buffer methods or fixed-threshold models, its smoothness helps avoid boundary effects, better reflecting actual tourist decision-making behavior and is suitable for continuous spatial analysis. In addition, the Gaussian decay function can integrate multi-source data to achieve the collaborative optimization of fairness and efficiency. In this study, the Gaussian function was selected as the distance decay function for 2SFCA. It utilizes the area of rural tourism sites as the supply scale and the population of demand points as the demand scale. To reflect the impact of rural tourism sites on residents’ willingness to travel, different search radii $d_{\text{0}}$ are set according to the scale and popularity of the rural tourism sites. The larger the scale and popularity, the more residents are willing to travel long distances to visit, and the larger the search radius will be. Parameter settings are illustrated in Fig 3.

K-means remains widely used today because of its simplicity and ease of implementation. As a type of clustering analysis method, it is often used to determine quantities of clusters. The quality of the clustering is assessed by the magnitude of the sum of squared errors of the points within each cluster relative to the cluster center, with a smaller sum of squared errors indicating a more effective clustering outcome. The number of clusters must be determined in advance. If the number of clusters is too small, it will result in large differences within the cluster; if the number of clusters is too large, it will result in small differences between clusters. In 1998, Rezaee proposed that the optimal number of clusters is between [1, n], where n is the size of the dataset [42].

#### Rural tourism facility location and capacity optimization model.

The P-median model selects suitable locations for facilities based on the specific number of facilities. This minimizes the total distance from demand points to facility points, thereby achieving the lowest overall travel cost of the location layout. It is also known as the minimization of impedance model. The mathematical formulas can be seen in Table 1.

The Maximum Coverage Model (MCM) is used when the number of facility points has been selected. Suitable locations for the facility points are chosen so that the maximum number of demand points within a certain service range are covered [43]. Particle Swarm Optimization (PSO) is an algorithm that originated from studying the foraging behaviors of flocks of birds, swarms of insects, and schools of fish [44]. This paper aims to minimize the total distance between particles with poor accessibility by using PSO as the optimization objective function. It incorporates eight potential development areas as the particle swarm scale, iteratively updating particle positions through the particle swarm optimization process. This results in the calculation of the optimal local solution of the particles and the optimal global solution of the particle swarm. Genetic Algorithm (GA) is a search heuristic algorithm that models computer systems based on biological evolution [45], making it suitable optimization problems.

## Results

### Accessibility analysis of the 2SFCA

Fig 1. displays the distribution of the actual overall accessibility to each community measured by the 2SFCA method. In Wuhan, the actual multimodal accessibility to communities indicates an uneven distribution pattern. High accessibility is observed primarily in the six principal suburbs (e.g., Xinzhou, Huangpi, Dongxihu, Caidian, Hannan, and Jiangxia). Particularly, the proximal accessibility of the main urban areas (e.g., Huangpi, Dongxihu, and Jiangxia) is significantly higher, due to their proximity to rural tourism destinations and higher aggregation of destinations. In contrast, accessibility in the main urban and suburban areas is relatively low.

Table 2 shows the number of accessible rural destinations and the average time to reach those destinations. The average amount of time to reach rural tourism sites shows considerable variation between suburbs and main districts. The proportion of suburbs to destinations is 76.41%, composing over three-fourths of the total destinations. In contrast, the main districts comprise just 23.59% of the accessible rural tourism destinations. Among the suburban districts, Huangpi holds the largest share at 20.49%, while Hannan has the smallest at 1.37%. The remaining districts each contribute over 10%. The average travelling time is 15.67 minutes for suburbs and 24.14 minutes for the main cities. The distribution of rural tourism destinations is generally uneven, with the suburban area markedly higher than the central area in synthetic accessibility, with blind zones in every district. Nonetheless, further optimization of rural tourism accessibility remains necessary.

**Table 2:** Table of reachable numbers and average times of rural tourism destinations in districts of Wuhan City.

Districts	Amount of accessible rural tourism destinations	Proportion/*%*	Average time to reach rural tourism destinations/*min*
Hanyang	6,449	3.58	22.51
Jiangan	8,755	4.87	21.00
Jianghan	5,486	3.05	20.93
Qiaokou	7,501	4.17	21.66
Wuchang	5,517	3.07	26.86
Hongshan	6,558	3.64	25.64
Qingshan	2,066	1.10	30.40
Xinzhou	22,724	12.64	15.09
Dongxihu	20,853	11.6	13.10
Caidian	31,702	17.64	15.78
Jiangxia	22,771	12.67	16.54
Huangpi	36,835	20.49	15.38
Hannan	2,473	1.37	18.14
Total	179,690	100	17.19

### Quantity-location of optimized sites based on multiple models

This study employed K-means model to confirm the number of additional locations. We isolated 2851 units of data by assigning accessibility values to each community, dividing accessibility into seven levels based on natural breaks, and extracting the two lowest levels of data (accessibility values<357). Through repeated experiments, we predetermined the maximum quantity to be 80, then used MATLAB programming to implement the K-means model. The results of the iterative experiment revealed that as the number of clusters increases, the average furthest distance sharply decreases where the number of clusters is [1,8]. The trend slows and remains unchanged after node 8. Based on the experimental results and the actual constraints of land development costs, we selected 8 as the number of additional tourist locations to achieve a balance between cost and benefit.

The new location selections of the three models are shown in Fig 5. The P-median highlighted addresses in Huangpi, Dongxihu, Jiangxia, Caidian, and Xinzhou. The addresses selected by the MLM are in Dongxihu District, Jiangxia District, Caidian District, and Xinzhou District. Additionally, the addresses selected by the PSO are distributed across Huangpi, Jiangxia, Caidian, and Xinzhou.

**Fig 4:**
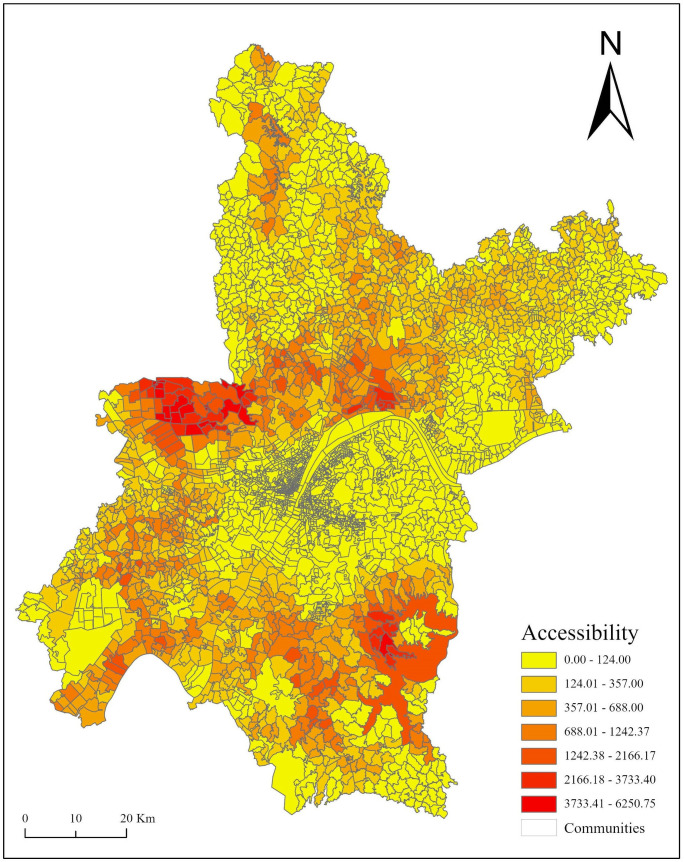
The distribution of rural tourism accessibility in Wuhan City.

**Fig 5:**
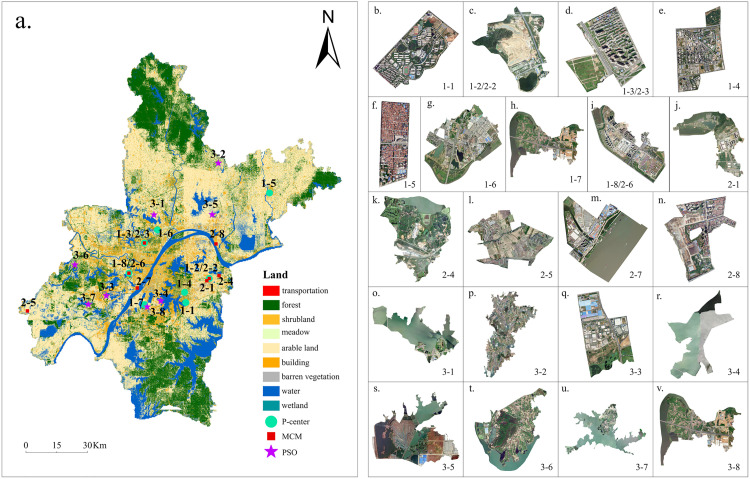
Location and remote sensing images of new tourism destinations (1-P-median, 2-MCM, 3-PSO).

Based on the overall site selection simulation results from the three models, the potential candidates for rural tourism are consistently distributed around the main urban areas in Wuhan. This may be due to the placement of rural tourism sites as facility points predominantly within the suburban districts of Wuhan. However, some of the community resident points, serving as demand points, are situated in the main urban area. Consequently, the calculations for the three models must be closely aligned with the main urban area to fulfill the requirements of each objective function. Individually, there are differences in the candidate selection sites for the three models. The results of the P-median model are almost adjacent to the main urban area (except for location 1–5). This is due to Xinzhou’s accessibility being generally insufficient, compared to other outlying districts. Moreover, the distance between the demand points and facility points in Xinzhou also require optimization. Due to this, Location 1–5 is situated in the central part of Xinzhou, rather than near the main urban area. The results of the MLM are almost entirely restricted to the main urban area (with the exception of Location 2–5). To address the needs of southwestern communities, where rural tourism destinations are limited, Location 2–5 in Caidian was chosen. Nevertheless, the PSO computational results are relatively well-dispersed because the selected areas in the PSO reflect the diverse needs of the community. These points are not easily accessible, and the candidate locations are distributed at many positions within the six suburban districts.

To determine the potential locations, research must maximize social benefits and consider the actual situation of Wuhan’s urban construction. To verify the theoretically optimal locations derived from the aforementioned models, this study carries out a comprehensive study on the suitability of potential development areas. This is based on the land use and remote sensing images of Wuhan and accounts for the surrounding environment, the degree of resource availability, and the population distribution. The analysis results are shown in Fig 4 and Table 2. Table 2 demonstrates that in Locations 1–2 (same as 2–2), 2–1, 2–4, 2–5, 2–7, 3–1, 3–2, and 3–4, where land and water resources are relatively abundant, measures can be adopted which address local conditions and optimize and integrate existing resources to build new rural tourism sites in Wuhan. In Locations 1–1, 1–3 (same as 2–3), 1–4, 1–5, 1–6, 1–7, 1–8 (same as 2–6), 2–8, 3–3, 3–6, and 3–8, where land resources are tight, upgrades and renovations can be implemented according to the individual situations of the locations to provide rest and relaxation functions. In Locations 3–5 and 3–8, enhancements to the ecological environment of rivers and lakes, as well as improvements to the water quality, can lead to greater ecological and social benefits. The final determination of the optimal potential development area locations includes 1–2 (same as 2–2), 2–1, 2–4, 2–5, 2–7, 3–1, 3–2, and 3–4.

### Determination of the capacity of potential development areas

As rural tourism rapidly expands, addressing efficiency and equity issues has become increasingly urgent. A suitability analysis of potential rural tourism development areas in Wuhan was conducted, integrating remote sensing imagery and land use data for the city. This approach was based on the number of identified potential development sites. Eight resource-rich sites were identified as alternative locations. Next, the service capacity of potential development areas was determined using a genetic algorithm to calculate the minimum difference in accessibility for community residents to rural tourism sites. This algorithm primarily considers the spatial heterogeneity of demand-side factors (population density, existing facility coverage), assuming the initial capacity of new tourist sites as the average service capacity of existing scenic areas. It then reallocates the capacity of each point to achieve a supply-demand balance. This completion signifies the conclusion of the second step in the two-step optimization process aimed at improving spatial accessibility, thereby enhancing equity. This study chose the average service capacity of the existing scenic area (619,876.86) as the initial capacity for the eight potential development areas Table 3. The final optimization results are shown in Table 4.

**Table 3:** New location capacity and proportion.

Location	Primitive capacity	Optimized capacity	Proportion/%
1-2	619,876.86	640,192.13	12.91
2−1	619,876.86	578,904.10	11.67
2-4	619,876.86	575,745.77	11.61
2-5	619,876.86	8,070.25	0.16
2-7	619,876.86	512,835.44	10.34
3−1	619,876.86	1,711,459.36	34.51
3−2	619,876.86	470,213.48	9.48
3-4	619,876.86	461,579.47	9.31

**Table 4:** New tourism destination environmental description of P-median, MLM, and PSO.

Model	Location	Lon	Lat	Environment
P-Median	1−1	114.43	30.42	Next to the school building and dormitory; Less unused land
	1-2	114.53	30.51	South of a park; Empty with fewer buildings and ample land resource
	1-3	114.25	30.65	Near the highway; Residential neighborhoods and scarce land resources in the east side
	1-4	114.42	30.46	Next to the school building and dormitory; Less unused land
	1-5	114.80	30.84	Next to a shopping mall; Clustered buildings, with scarce vacant land
	1-6	114.31	30.70	Dense transportation network; Near neighborhoods; Scarce vacant land
	1-7	114.25	30.41	Close to the highway and elementary school; Dense transportation; Less unused land resources
	1-8	114.18	30.53	Near an industrial area; Dense buildings and transportation; Less available land
MLM	2−1	114.52	30.50	Located on the east side of a park; Few buildings and more vacant land resources
	2−2	114.53	30.51	Same as 1–2
	2-3	114.25	30.65	Same as 1–3
	2-4	114.57	30.52	Near a company with abundant water resources and sparse buildings; Adequate land resources
	2-5	113.74	30.39	Southeast of a highway; Few buildings; Small wooded areas and abundant land resources
	2-6	114.18	30.53	Same as 1–8
	2-7	114.22	30.48	Located on the east of the Yangtze River; Most enterprise company nearby; More available land
	2-8	114.56	30.64	Nearby a company with factory buildings; Less available land
PSO	3−1	114.29	30.75	Located on the northeast of a lake, with abundant water resources and unused land
	3−2	114.57	30.95	Villages to the northeast; Scattered woods; More vacant land
	3−3	114.08	30.45	Nearby the automobile company; Dense buildings and scarce land
	3-4	114.32	30.43	Located east of a lake, with rich water resources and adequate unused land
	3-5	114.54	30.75	Located on the northwest of a lake; No articulable land resources
	3-6	113.94	30.56	Located on the north of a zoom; Concentrated forests, with fewer buildings and less useful land
	3-7	114.00	30.41	Located on the east of a lake; No articulable land resources
	3-8	114.26	30.41	Located in an industry park; Mostly factories and companies; Scarce vacant land resources

According to Table 4, Location 3–1 possessed the greatest capacity, accounting for 34.51% after capacity optimization, followed by Location 1–2 (12.91%). The capacities allocated to Locations 2–1, 2–4, 3–2, and 3–4 are relatively similar. However, Location 2–5 had the lowest capacity, accounting for only 0.16%. The maximum accessibility value for rural tourism in Wuhan was 6,250.75, with the average value being 227.23 and the standard deviation being 491.15. Post-optimization, the maximum value is 1,781.14, the average value is 109.56, and the standard deviation is 197.61. The standard deviation of the accessibility to rural tourism in Wuhan decreased after optimization, achieving the optimization goal.

## Conclusions

The accessibility of rural tourism sites in Wuhan exhibits the characteristic of “high-around- low in the middle”. Overall, accessibility is not high, and the spatial distribution is uneven. Regionally, accessibility is highest in Dongxihu, followed by Hannan, with Xinzhou showing the lowest accessibility. The study found that key factors influencing rural tourism site accessibility primarily include distance decay and site capacity.

This study combines theory with practice to optimize rural tourism destinations. By conducting a site selection simulation for potential development areas of rural tourism sites through the three steps of quantity, location, and capacity, the accessibility of rural tourism sites in Wuhan can be significantly enhanced. First, eight potential development areas were determined to be optimal through the clustering analysis method and the assessment of social benefits. Second, three site selection simulations conducted using the P-median, MLM, and PSO models revealed that the candidate point schemes for potential development areas of rural tourism sites in Wuhan are largely consistent, with all being primarily distributed around the main urban area. Eight rural tourism destinations were selected based on a comprehensive set of factors. Third, the accessibility of the main urban area was improved, and the overall standard deviation of accessibility significantly decreased after capacity allocation. Furthermore, the degree of inequity in accessibility was reduced, and the efficiency and equity of resource utilization was enhanced.

Overall, this study focuses on the three-step optimization practice method and ultimately achieves the optimization of tourism destinations, provides a methodological reference for future studies on rural tourism destinations, and our research results detail policies for the development of Wuhan City’s rural tourism.

## Discussion

This article theoretically and practically optimized rural tourism destinations. In terms of research methodology, this study integrates the P-median, MCM, and PSO models, which can more comprehensively coordinate efficiency and fairness compared to single facility location models [27–29]. For example, Tian et al. [34] focused solely on accessibility in hospital optimization, whereas this study further reduces the standard deviation (from 491.15 to 197.61) through capacity allocation. In this case, the new point capacity in Wuhan exhibits a unique “high in the periphery, low in the center” pattern. Compared to international contexts, the high population density in Asia results in a higher “fairness optimization weight” than in Europe and America. The research findings further reveal a significant spatial correlation between the optimization results and the distribution of local cultural resources. High-capacity sites (such as 3–1) are located in areas rich in cultural resources, while low-capacity sites (such as 2–5) are situated in emerging urbanized regions. Among the eight newly added locations, five are adjacent to water bodies or woodlands, which is similar to the findings in study 7, reflecting tourists’ preference for a “natural escape experience” [7].

### Policy implications

Additionally, it should be noted that improving the infrastructure and service quality of rural tourism destinations is equally critical. Locations with lower capacity in the optimization results (such as 2–5) may require prioritizing the upgrade of existing facilities rather than new construction. Therefore, targeted policies are proposed based on the results.①Tiered development strategy: prioritize investment in high-capacity points (such as 3–1) and coordinate upgrades and optimization of existing facilities at low-capacity points (such as 2–5).②Ecological-cultural synergy: further strengthen ecological protection at points adjacent to water bodies or forests.③Infrastructure support enhancement: optimize public transportation routes to cover accessibility blind spots (such as Qingshan).④Establish a “dynamic-assessment-feedback” mechanism to adjust facility functions based on visitor satisfaction.

### Research limitations

Despite these contributions, there are still limitations. Firstly, the research mainly focuses on optimizing spatial accessibility (efficiency and equity), with less attention given to the optimization of non-spatial dimensions such as the specific types and characteristics of rural tourist sites, cultural connotations, service quality, and visitor satisfaction. However, these factors are also crucial for the sustainable development of rural tourism. Secondly, the model assumptions are relatively simplified. The capacity optimization model (GA) aims to minimize accessibility disparities (improving equity) as its main objective and assumes an initial capacity, but does not delve into how real-world constraints such as land costs, construction costs, operation and maintenance costs, and environmental impacts affect optimal capacity. The optimization results serve more as theoretical references for spatial configuration. Finally, the research optimizes based on the current transportation network and population distribution, but the attractiveness and capacity of rural tourist sites may change over time (such as seasonal fluctuations, facility aging or upgrades). In the future, the research model can fully consider factors such as urban expansion, transportation development, population migration, and visitor preference changes for dynamic simulation.

## Supporting information

S1 Data Code. (ZIP)
